# Evaluating the risk of SARS-CoV-2 reinfection with the Omicron or Delta variant in Wales, UK

**DOI:** 10.1371/journal.pone.0309645

**Published:** 2024-09-06

**Authors:** Mark Postans, Nicole Pacchiarini, Jiao Song, Simon Cottrell, Catie Williams, Andrew Beazer, Catherine Moore, Thomas R. Connor, Christopher Williams

**Affiliations:** 1 Communicable Disease Surveillance Centre (CDSC), Public Health Wales, Cardiff, Wales, United Kingdom; 2 Pathogen Genomics Unit, Public Health Wales, Cardiff, Wales, United Kingdom; 3 Wales Specialist Virology Centre, Microbiology, Public Health Wales, Cardiff, Wales, United Kingdom; 4 Cardiff University School of Biosciences, Cardiff University, Wales, United Kingdom; Gabriele d’Annunzio University of Chieti and Pescara: Universita degli Studi Gabriele d’Annunzio Chieti Pescara, ITALY

## Abstract

Recent studies suggest an increased risk of reinfection with the SARS-CoV-2 Omicron variant compared with previous variants, potentially due to an increased ability to escape immunity specific to older variants, high antigenic divergence of Omicron from earlier virus variants as well as its altered cell entry pathway. The present study sought to investigate epidemiological evidence for differential SARS-CoV-2 reinfection intervals and incidence rates for the Delta versus Omicron variants within Wales. Reinfections in Wales up to February 2022 were defined using genotyping and whole genome sequencing. The median inter-infection intervals for Delta and Omicron were 226 and 192 days, respectively. An incidence rate ratio of 2.17 for reinfection with Omicron compared to Delta was estimated using a conditional Poisson model, which accounted for several factors including sample collection date, age group, area of residence, vaccination and travel status. These findings are consistent with an increased risk of reinfection with the Omicron variant, and highlight the value of monitoring emerging variants that have the potential for causing further waves of cases.

## Introduction

SARS-CoV-2, the causative agent of coronavirus disease 2019 (COVID-19), has developed an increasingly genetically diverse population, which is monitored in Wales for signs of increased transmission and infectiousness [1]. As at 9 February 2023, 896,264 cumulative laboratory-confirmed cases of SARS-CoV-2 have been reported in Wales. Over the course of the COVID-19 pandemic, the UK saw a set of waves of infection, dominated by viruses from specific genetic backgrounds [2]. The first case of COVID-19 was reported in late February 2020 [3], and the original Wuhan reference strain circulated (GenBank accession number MN908947) [4], followed by a wave of cases that peaked in April 2020. A second wave, driven largely by a variant of the B.1 lineage containing the D614G mutation [5], re-established SARS-CoV-2 transmission in Wales in the Autumn of 2020, and precipitated a firebreak lockdown in October 2020. The emergence of the Alpha variant (pango lineage B.1.1.7) in late November 2020 [6], saw a third wave of infections, which peaked in January 2021. The fourth wave was dominated by the Delta variant (pango lineage B.1.617.2) [7], with the first cases being reported in Spring 2021, and a peak in October 2021. The final, fifth wave was driven by the Omicron variant (pango lineage B.1.1.529) [8], which established itself in November 2021 and subsequently became the dominant circulating lineage in Wales. Since then, the descendents of Omicron have diversified further, and Omicron is the parental lineage of the main current circulating SARS-CoV-2 variants in Wales [9, 10].

The Omicron variant led to a substantial rise in COVID-19 cases, many of which were reinfections due to the capability of the Omicron variant to escape immunity gained from previous natural infection and/or vaccination [11–14]. A number of studies have reported that reinfections are not common and the rate of reinfection differs between countries with estimates ranging from <0.5%-5% [15–18]. However, recent studies have also indicated an increased risk of reinfection with the Omicron variant compared with previous variants [19–21]. It is hypothesised that this is due to the Omicron variant having an increased ability to escape immunity specific to previous SARS-CoV-2 variants [22, 23]. Moreover, the Omicron variant has considerable antigenic divergence from the ancestral virus (featuring at least 50 new mutations) [24] and had a different entry pathway preference to the previous variants [25]. Preliminary work within the United Kingdom revealed that Omicron was associated with a 5.41 fold higher relative risk of reinfection compared with Delta [20]. This is consistent with previous analysis in in South Africa whereby they report a substantial increase in the risk of reinfection during the emergence of Omicron and not during periods where the Beta and Delta variants were dominant [19]. It is important to monitor the incidence and risk of reinfection, particularly as new and emerging SARS-CoV-2 variants arise. Specifically, this should be done in the context of protection from natural infection and/or vaccination in order to guide management and policy measures. The present study sought to investigate epidemiological evidence for differential SARS-CoV-2 reinfection intervals and incidence rates for two recent ‘wave-causing’ variants within Wales: Delta and Omicron.

## Methods

### Ethics

The study presented encompasses two elements. The first of these does not require specific ethical approval, as it focuses on public health/surveillance questions that make use of sequence data and other metadata that is already shared with the wider world as part of the activities of the COG-UK consortium (https://www.cogconsortium.uk/). COG-UK data is released and is publicly available via the ENA, GISAID and the COG-UK website. The element of the work that would/could require ethical approval is the specific examination of outcome data. The use of named patient data in the investigation of communicable disease outbreaks and surveillance of notifiable disease is permitted under Public Health Wales’ Establishment Order. Data were held and processed under Public Health Wales’ information governance arrangements, in compliance with the Data Protection Act, Caldicott Principles and Public Health Wales guidance on the release of small numbers. No data identifying protected characteristics of an individual were released outside Public Health Wales. The use of the genomic dataset for research purposes is also covered as part of the COG-UK project protocol which was approved by the Public Health England Research Support and Governance Office (RSGO) following review by the PHE Research Ethics and Governance Group (REGG).

### Data

The Genomic Epidemiology Team within the Public Health Wales Communicable Disease Surveillance Centre maintain a dataset that combines testing data from individuals identified as infected with SARS-CoV-2 with information about the variant implicated based on subsequent genomic sequencing or genotyping. This record-level surveillance dataset, which necessarily contains information enabling identification of individual cases post data-collection, was accessed by the research team on 09/06/2022 for the purposes of the retrospective study reported below.

All SARS-CoV-2 real-time PCR testing was conducted in line with local guidelines. Samples came from two sources. One set of samples (N = 65262, 25.97%) came from the NHS Wales laboratory network. These samples were predominantly hospital and staff samples, as well as some community cases. Of these samples, Reflex PCR testing was undertaken on 11036 samples (16.91%) to identify key spike mutations associated with VOC/VUIs using the Allplex SARS-CoV-2 Variant PCR assays (Seegene, Seoul, Korea) and subsequently sent for confirmatory WGS. The second set (N = 183924, 73.18%) of samples came from the lighthouse lab based in Newport, Wales, operated by Perkin Elmer, and are predominantly community cases, tested as part of the national Test, Trace and Protect programme. Lighthouse lab diagnostic residual samples were collected by PHW, and the Public Health Wales Pathogen Genomics team undertook cherry-picking of positive samples for sequencing. The origin of a further 2143 (0.85%) of samples was not readily coded but these were also available for analysis.

Regardless of sample origin, the Public Health Wales Pathogen Genomics Unit aimed to sequence all samples that were of sufficient quality, as measured by a low qPCR cycle threshold (CT). The threshold to accept a sample for sequencing was generally ≤ 29.99 [26]. However, this cutoff varied throughout the study period due to changes in testing demand and capacity. PCR amplicons were generated using the Illimina COVID-Seq kit with ARTIC primers sourced from IDT. Samples were then loaded onto Illumina NextSeq 550 systems using a 300 cycle medium or high output kit. Once sequences were generated, these were processed by the Nextflow ARTIC nCoV pipeline (see https://github.com/connor-lab/ncov2019-artic-nf). Variants were identified using the software aln2type (see https://github.com/connor-lab/aln2type) using the UKHSA variant definitions (see https://github.com/ukhsa-collaboration/variant_definitions). Assembled genomes and bams were then filtered for human reads and uploaded with metadata to CLIMB COVID, the UK SARS-CoV-2 data analysis platform established by COG-UK [27, 28] to allow UK-wide collation, management and processing. Sequences were subsequently analysed using the CLIMB COVID phylogenetics pipeline which assigns cases to a PANGO lineage [28] and a putative ‘UK transmission group’ using ancestral state reconstruction [29, 30]. PANGO lineages were mapped to WHO variant of concern labels (see https://github.com/phe-genomics/variant_definitions; accessed 12/04/2023). Samples designated as ‘Wildtype’ according to this WHO labels classification scheme were relabeled as ‘Wuhan-Hu-1’. The results from the analysis were then synthesized into a genomics line list, and reported to the Public Health Wales Communicable Disease Surveillance Centre, for integration into a larger combined dataset.

The Wales Immunisation System was used to ascertain vaccination status of confirmed cases. An individual was considered to be “vaccinated” [with 2 doses] if they had had two doses of vaccine 14 days prior to their sample date. ICNet was used to identify case admissions to hospital. ICNet is a hospital infection prevention case management and reporting system used across Wales by infection prevention and control (IPC) teams and for systematic surveillance by Public Health Wales. An admission was classified as an individual with a positive PCR result for COVID-19, who was admitted to hospital on or one day before the day of their first positive test, or in the 28 days following a positive test.

Records for all genotyped and sequenced samples that were collected up to and including February 2022 were extracted for analysis. Note that there is no WHO variant classification for the original wild type SARS-CoV-2 virus as this was not, by definition, a ‘variant’. Therefore, all records without a WHO variant classification and which were collected prior to 22nd March 2021, were presumed to be ‘Wuhan-Hu-1’ infection cases (note that this corresponds to the WHO ‘Wildtype’ classification). Where a variant classification was not available for samples collected after this date, the classification is considered indeterminate or otherwise missing and therefore the corresponding case record was not included in our subsequent analyses. Records that were not dated were also excluded, as were records with no available date of birth.

Probabilistic matching based on the forename, surname, date of birth and postcode fields of each record was then performed using the fastLink package v0.6.0 in R [31], to identify individuals with multiple records in the dataset. The default string match threshold of 0.94 was applied. For the present work, a reinfection was defined as a second positive test record with a corresponding WHO classification, occurring at least 42 days after the first positive test recorded for the same individual. This was consistent with the standard 42 day episode deduplication rule that was used for SARS-CoV-2 surveillance over the study period by Public Health Wales, and is comparable to the interval used in other recent reinfection studies [32]. The matched record pairs that satisfied these criteria were visually inspected and any clearly erroneous matches were removed prior to analysis.

### Omicron versus Delta reinfection frequency and inter-infection interval

The time series of the weekly number of positive samples recorded up to 28th February 2022 was first plotted by both week and WHO classification label (Alpha, Beta, Delta, Eta, Gamma, Kappa, Omicron, Zeta, Wuhan-Hu-1, and Unknown), alongside the log-transformed number of cases reported in Wales (case data publicly available at https://coronavirus.data.gov.uk/details/cases?areaType=nation&areaName=Wales, accessed: 10/03/2023). The rolling 7-day average number of samples is also plotted by both sample date and infection type (first versus second infection) to illustrate the temporal distribution of the number of first versus second infections.

Case counts and proportions were then tabulated by first and second infection variant to a) examine the frequency of Omicron versus Delta reinfection, and b) assess whether individuals were more likely to be infected first with the Wuhan-Hu-1, Alpha, Delta or Omicron variants. A chi-square test of independence was applied to the proportions in the resulting 2 (second infection: Delta or Omicron) x 4 (first infection: Wuhan-Hu-1, Alpha, Delta, or Omicron) contingency table to assess whether the first infection variant was independent of the second infection variant. The proportions and chi-square test are also illustrated on a mosaic plot alongside Pearson residuals to indicate the contingency table cells contributing to the overall test statistic. The mosaic plot was created using the VCD package v1.4–10 in R [33]. A Cramer’s V statistic was computed to estimate the Chi-square effect size. Among other assumptions, the Chi-square test assumes that at least 80% of cells contain more than 5 cases and that no cell has an expected value lower than 1. Both assumptions were satisfied in this case although one cell did have a small expected value of approximately 2. A complementary Fisher’s exact test was therefore calculated (not shown), which corroborated the chi-square test result.

The difference in sample collection date for first and second infections was used as a proxy measure for the inter-infection interval. To assess whether there is a difference in reinfection interval between the Delta and Omicron variant, the mean, median, standard deviation, interquartile range, minimum and maximum inter-infection intervals were also tabulated by second infection variant. These descriptive statistics, together with frequency histograms and normal probability plots (not shown) indicated that the reinfection intervals were not normally distributed, a finding confirmed by a formal Shapiro Wilk normality test. A two-tailed Kruskal-Wallis test was therefore used to compare the reinfection intervals of the Delta and Omicron variants.

All inferential statistical tests were two-tailed and conducted using a significance threshold of p ≤ 0.05, unless otherwise stated.

### Characteristics and exposures of individuals reinfected with Omicron versus Delta

Descriptive epidemiology of the characteristics of confirmed Omicron reinfection cases was compared with confirmed Delta reinfection cases following tabulation of counts and proportions of both Delta and Omicron reinfection cases with each characteristic of interest. The characteristics considered included Local Health Board of Residence (Aneurin Bevan, Betsi Cadwaldr, Cardiff and Vale, Cwm Taf Morgannwg, Hywel Dda, Powys, or Swansea Bay), Age Group (0–24, 25–59, or 60+), Vaccination Status (Unvaccinated, One Dose, Two Doses, Three Doses, or Unknown), recent international Travel status (Yes or No), Symptom Status (Symptomatic, Asymtomatic or Unknown) and Hospitalisation Status (Hospitalised, Not Hospitalised or Unknown).

To investigate the effect of these characteristics and exposures upon the odds of reinfection with Omicron as opposed to Delta, an additional binary variable denoting Omicron reinfection status was derived (1 = reinfected with Omicron, 0 = reinfected with Delta). This binary response was regressed on local health board, age group, vaccination and travel status, using binary logistic regression with a canonical logit link. Age group, vaccination and travel status were of a priori interest based on previous research highlighting these as infection or reinfection risk factors [34–37]. As health service planning and delivery is the responsibility of seven independent Local Health Boards in Wales [38], cases’ Local Health Board of residence was also included as a predictor to account for any geographical variation in healthcare delivery and outcomes that may be germane to SARS-CoV-2 reinfection risk, and other spatial covariates and confounds which may be difficult to directly measure. Symptom and hospitalisation status were not included as predictors because the corresponding database fields were very sparsely completed.

Model fit statistics including residual deviance and degrees of freedom are reported. Odds ratios (ORs) were also computed alongside the corresponding p-values and 95% confidence intervals (CIs). A statistical significance threshold of p ≤ 0.05 was applied.

We also fit a conditional Poisson regression model [39] to estimate the difference in reinfection rates between the Omicron and Delta variants, accounting for variation in the sample collection date as well as key demographic characteristics and exposures. Reinfection counts were regressed on second infection variant (Omicron versus Delta) using a conditional Poisson model with a canonical log-link, implemented using the gnm package v1.1–2 in R [40]. Rather than including additional model terms to account for other factors and personal characteristics that may influence reinfection (e.g., specimen date and age group), and estimating separate coefficients for those variables as per unconditional Poisson regression, in the conditional Poisson model we can condition on the sum of events occurring in each of the model strata. The strata are determined by the interaction of the other variables, the effects of which are thus ‘conditioned out’. Here, the model strata were implemented as the interaction between the date that the second positive sample was collected, local health board, age group, vaccination and travel status. Note that as the second sample collection date was included in this interaction, overall infection pressure, testing policy etc., were effectively held constant for the Delta and Omicron reinfection cases being compared within the model strata. Again, symptom and hospitalisation status were not included in the model strata because the corresponding database fields were very sparsely completed. The point estimate and 95% CI for the incidence rate ratio is reported, alongside the model residual deviance and degrees of freedom.

## Results

### Omicron versus Delta reinfection frequency and inter-infection interval

**Fig 1:**
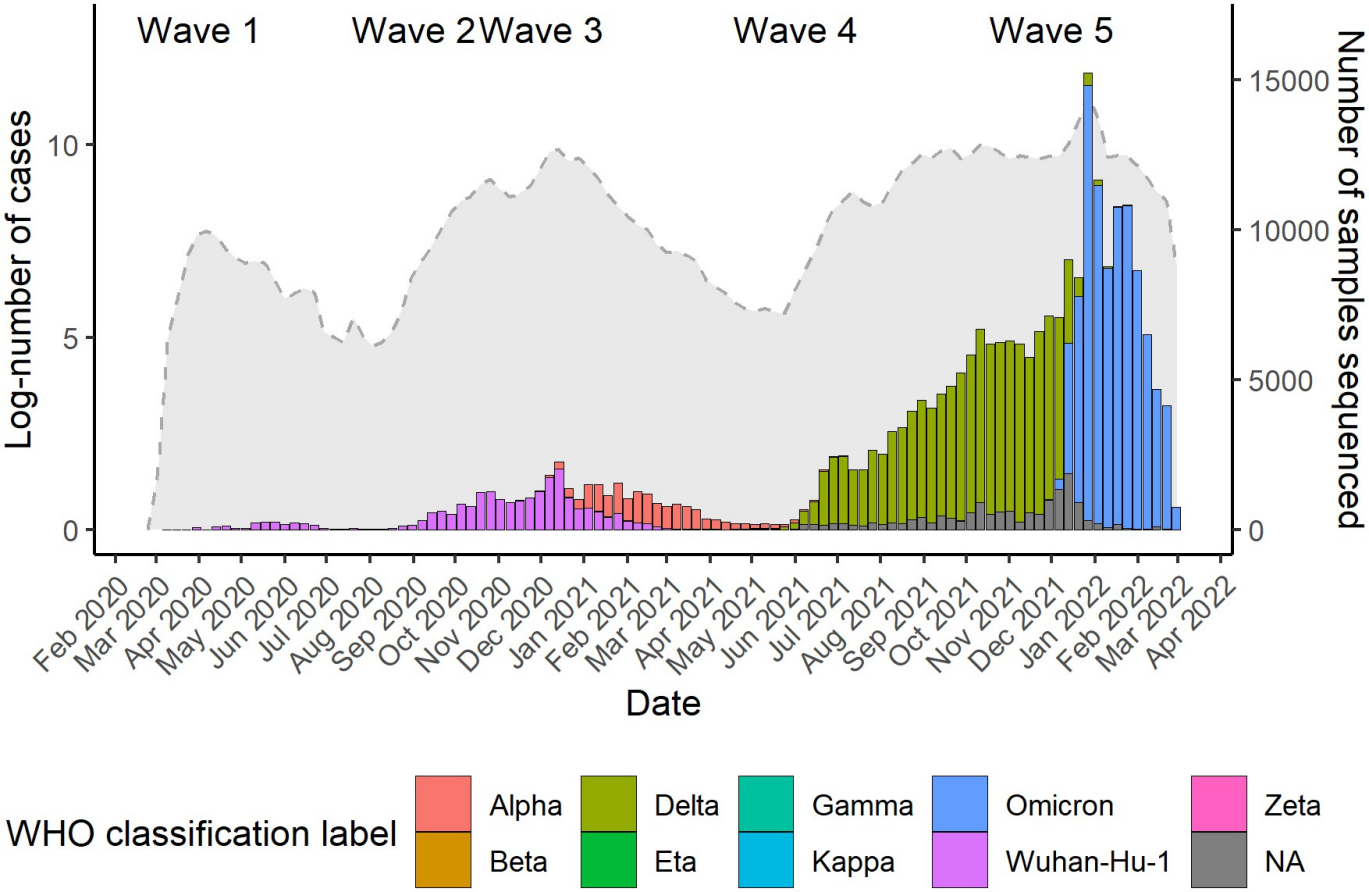
Weekly positive samples sequenced by WHO classification label (stacked bars) and the log-transformed number of cases in Wales (gray shaded area). A total of 251,329 positive samples were sequenced or genotyped over the study period.

Consistent with other UK nations, Wales experienced a first wave of cases attributable to the original SARS-CoV-2 strain, which peaked in April 2020. A second wave of cases commencing in Autumn 2020 was driven by a variant of the B.1 lineage containing the D614G mutation. Henceforth we refer to both of these early variant groups as ‘Wuhan-Hu-1’, which corresponds to the WHO ‘Wildtype’ classification label. Note that testing capacity, and by extension, whole genome sequencing and genotyping capacity, was limited during these early waves of cases compared to subsequent waves. A third wave of cases coincided with the dominance of the Alpha variant from January 2021. A fourth wave of cases began in June 2021, at which point the Delta variant became dominant until the Omicron variant caused a fifth wave of record infections in December 2021 (Fig 1).

**Fig 2:**
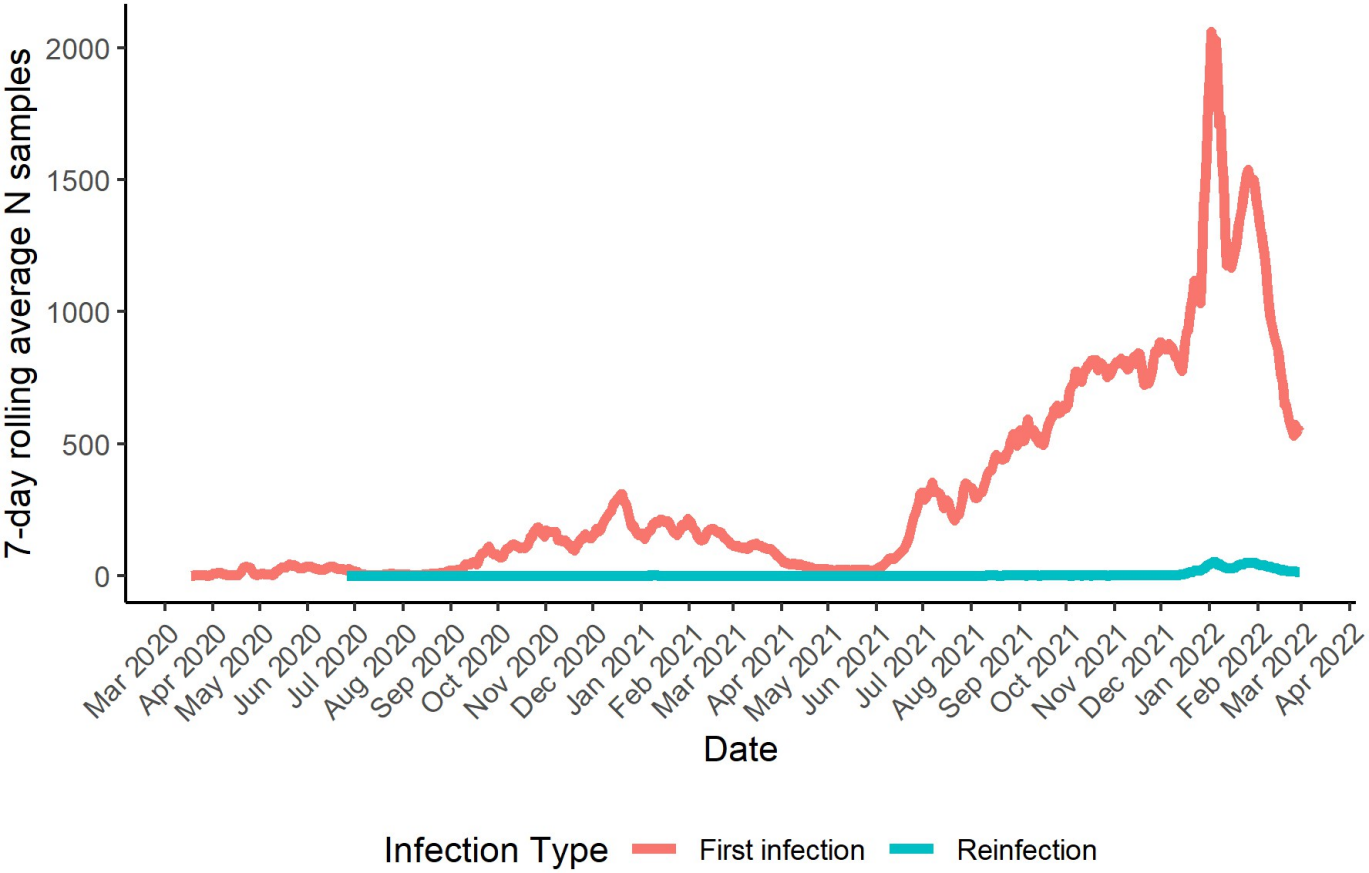
Rolling 7-day average number of samples by sample date and infection type.

Fig 2 shows the 7-day rolling average number of samples collected by infection type (initial infection versus reinfection), highlighting that the vast majority were initial infections. The minority of samples associated with a ‘reinfection’, as defined here, were evidently collected towards the end of the timeseries when the Omicron variant became dominant in the population.

A total of 2488 reinfection cases were thus identified, comprised of 180 Delta reinfections (86 Males, mean age: 35.6 (years); STD: 19.2) and 2308 Omicron reinfections (898 Males, mean age: 31.6 (years); STD: 18.4). For both groups, the majority of the reinfection samples were sourced from the lighthouse lab network, though the proportion was higher for Omicron compared to Delta cases (75% and 62%, respectively; see Table 1).

**Table 1:** Delta and Omicron reinfection counts and percentages by sample origin. Percentages computed by column.

	Second infection			
	Delta	Omicron	Total	p-value^1^
Sample origin				<0.001
NHS labs	59 (33%)	546 (24%)	605 (24%)	
Lighthouse labs	111 (62%)	1,738 (75%)	1,849 (74%)	
Unknown	10 (5.6%)	24 (1.0%)	34 (1.4%)	
Total	180 (100%)	2,308 (100%)	2,488 (100%)	

^1^Pearson’s Chi-squared test

The reinfection cases were distributed across the first and second infection variants considered here, as shown in Table 2. For those individuals reinfected with the Delta variant, the most frequent initial infection was Wuhan-Hu-1 SARS-CoV-2 (45.6%) whereas for those reinfected with Omicron, the most frequent initial infection was the Delta variant (55.2%). One of the cases with an initial Wuhan-Hu-1 infection followed by a Delta reinfection, experienced a subsequent third infection with the Omicron variant, confirmed by an additional positive sample.

**Table 2:** Delta and Omicron reinfection counts and percentages by first infection variant. Percentages computed by column.

	Second infection			
	Delta	Omicron	Total	p-value^1^
First infection				<0.001
Delta	71 (39%)	1,273 (55%)	1,344 (54%)	
Wuhan-Hu-1	82 (46%)	730 (32%)	812 (33%)	
Alpha	27 (15%)	282 (12%)	309 (12%)	
Omicron	0 (0%)	23 (1.0%)	23 (0.9%)	
Total	180 (100%)	2,308 (100%)	2,488 (100%)	

^1^Pearson’s Chi-squared test

**Fig 3:**
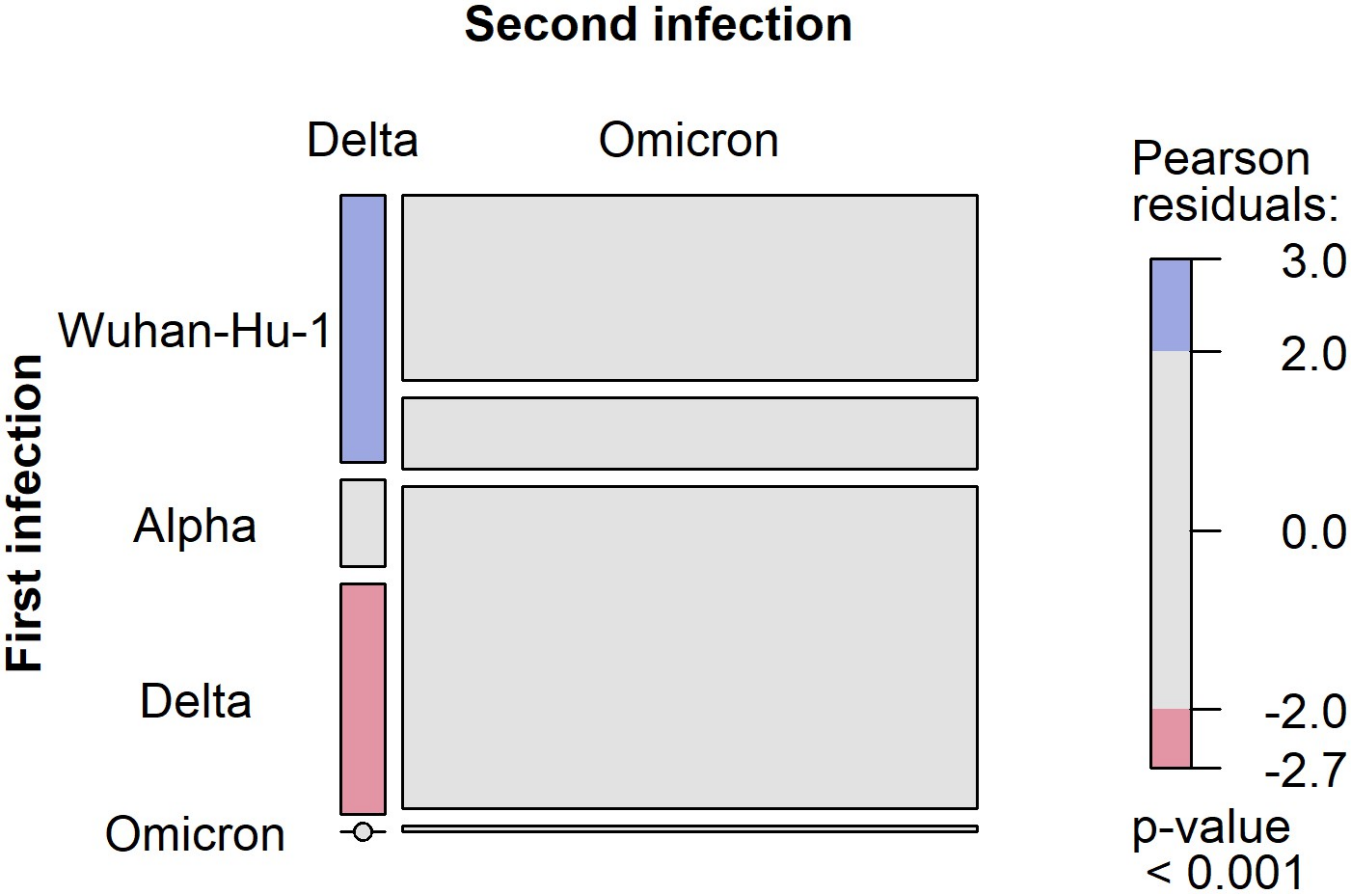
Mosaic plot of the number of reinfections by first infection and second infection variant. The area of each tile is proportional to the corresponding contingency table cell count in Table 1. The vertical height of each tile in the first (second) column reflects the proportion of Delta (Omicron) reinfections that were associated with an initial Wuhan-Hu-1, Alpha, Delta or Omicron infection. The tile shading represents the magnitude and sign of the Pearson residual for the cell of the associated contingency table. Red indicates a cell with fewer cases than expected whereas blue indicates a cell with more cases than expected under the null model.

A Chi-square independence test confirmed that the proportions of individuals infected with each of the four first infection variants considered here (Wuhan-Hu-1, Alpha, Delta or Omicron), differed between the Delta and Omicron reinfection groups. (*X*^2^ (3, N = 2488) = 20.387, p ≤ 0.001). This effect is also evidenced in Fig 3, the shading of which indicates that individuals reinfected with Delta were more likely to have had an initial Wuhan-Hu-1 infection compared to those individuals reinfected with Omicron. They were also less likely to have had an initial Delta infection compared to those reinfected with Omicron. A small Cramer’s V statistic, however, indicates that this first by second infection variant effect was small (*V* = 0.091, 95% CI = [0.057, 0.131]).

**Fig 4:**
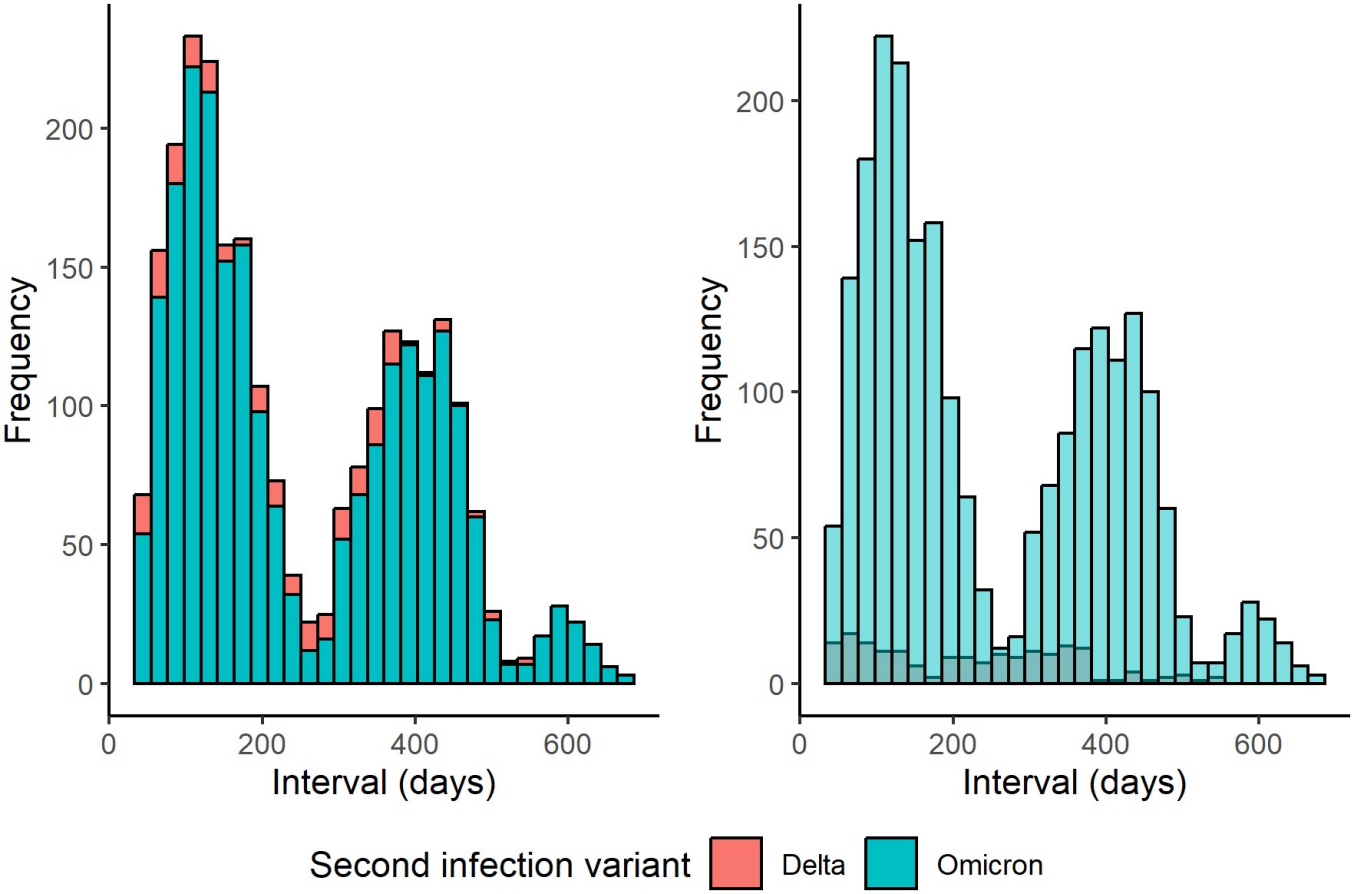
First-to-second COVID infection interval by second infection variant. Stacked and un-stacked histograms (left and right, respectively).

Turning to the inter-infection intervals, Table 3 shows the mean, median, standard deviation, standard error, interquartile range, and both the minimum and maximum inter-infection intervals for individuals reinfected with the Delta and Omicron variants. The distribution of these inter-infection intervals is also shown by reinfection variant in Fig 4. The median reinfection interval is shorter for the Omicron variant, consistent with a lower number of days between first and second infection compared to Delta. Note, however, that both the mean and maximum interval are larger for Omicron relative to Delta (as are the standard deviation and interquartile range), indicative of positive skew in the distribution of reinfection intervals for Omicron. This is arguably consistent with the timeline of the emergence of the Delta and Omicron variants. As Omicron emerged later, the maximum possible inter-infection interval is necessarily longer for Omicron compared to Delta reinfections.

**Table 3:** Inter-infection intervals (days) by second infection variant.

	Re-infection variant	
Reinfection Interval (days)	Delta	Omicron
Mean	223.1	253.8
Median	226.0	192.0
Std Deviation	131.3	156.0
Std Error of Mean	9.8	3.2
IQR	225.8	275.0
Min	42.0	42.0
Max	551.0	674.0

Indeed, formal statistical tests confirmed that a) the inter-infection intervals for both the Delta and Omicron reinfection groups were not normally distributed(*W* = 0.944, p = 0; *W* = 0.909, p = 0, respectively), and b) those reinfected with Omicron experienced fewer days between their first and second infection, compared to those reinfected with the Delta variant (*χ*^2^ (1) = 7.84, p = 0.005).

### Characteristics and exposures of individuals reinfected with Omicron versus Delta

A conditional Poisson model was used to estimate the incidence rate ratio for reinfection with the Delta versus Omicron variants based on event counts in the model strata (see Methods). The model had a residual deviance of 2.3 on 11 degrees of freedom, indicating a good fit to the data (*χ*^2^ (11) = 2.3, p = 0.997).

The estimated incidence rate ratio of 2.17 (95% CI = [1.12, 4.45]), indicates that when the other potentially important characteristics (e.g., sample collection date, age group and vaccination status) also accounted for in the model are held fixed, the Omicron variant was associated with approximately 2.17 times the number of reinfections compared with Delta (i.e., 116.7% more).

Table 4 below tabulates the counts and proportions of both Delta and Omicron cases with each level of a set of characteristics and exposures, including Local Health Board of Residence, Age Group, Vaccination Status, Travel Status, Symptom Status and Hospitalisation Status.

**Table 4:** Characteristics and exposures of Delta versus Omicron reinfection cases.

Characteristic	Delta, N = 180^1^	Omicron, N = 2,308^1^
Local Health Board		
Aneurin Bevan UHB	25 (14%)	376 (16%)
Betsi Cadwaldr University Health Board	44 (24%)	487 (21%)
Cardiff and Vale University Health Board	32 (18%)	440 (19%)
Cwm Taf Morgannwg University Health Board	25 (14%)	344 (15%)
Hywel Dda University Heath Board	15 (8.3%)	215 (9.3%)
Swansea Bay University Health Board	37 (21%)	402 (17%)
Powys Teaching Health Board	2 (1.1%)	43 (1.9%)
Unknown	0	1
Age Group		
0–24	57 (32%)	931 (40%)
25–59	101 (56%)	1,217 (53%)
60+	22 (12%)	160 (6.9%)
Vaccinated		
No	68 (38%)	756 (33%)
1 dose	12 (6.7%)	154 (6.7%)
2 doses	92 (51%)	1,325 (57%)
3 doses	1 (0.6%)	34 (1.5%)
Unknown	7 (3.9%)	39 (1.7%)
Travel	3 (1.7%)	43 (1.9%)
Symptomatic	5 (50%)	36 (75%)
Unknown	170	2,260
Hospitalised	1 (7.7%)	20 (24%)
Unknown	167	2,224

^1^n (%)

A multivariable logistic regression model was fitted to this data to investigate the effect of particular case characteristics on the odds of reinfection with Omicron as opposed to Delta. The fitted model, which included Local Health Board, Age Group, Vaccination status and Travel as predictors, provided an adequate fit to the observed data, with a residual deviance of 1261.38 on 2473 degrees of freedom (*χ*^2^ (2473) = 1261.38, p ≃ 1). Note that symptomatic and hospitalisation status were not included as predictor variables owing to the high proportion of records for which this information was missing (see Table 4 above). The computed odds ratios are reported in Table 5 below. For each characteristic (e.g., Local Health Board), the odds ratio reported at a given level (e.g., Powys Teaching Health Board) represents the relative odds of being reinfected with Omicron rather than Delta in the corresponding group of cases (i.e., Powys Teaching Health Board residents), compared to those in the reference group (i.e., Aneurin Bevan UHB residents), whilst all other characteristics included in the model are held fixed. An odds ratio (OR) > 1 indicates increased odds whereas an OR < 1 indicates decreased odds, compared to the reference group. Individuals aged 25–59 were therefore less likely to be reinfected with Omicron than Delta compared to those aged 0–24 years (OR = 0.54, 95% CI = [0.37, 0.78], p = 0.001). Likewise, individuals aged 60+ were also less likely to be reinfected with Omicron than Delta compared to those aged 0–24 years (OR = 0.26, 95% CI = [0.14, 0.47], p < 0.001). Finally, the odds of reinfection with Omicron rather than Delta was higher in individuals who received two doses of vaccine by the time of reinfection, compared to the unvaccinated (OR = 1.96, 95% CI = [1.33, 2.87], p < 0.001). All other odds ratios were not statistically significant.

**Table 5:** Characteristics and exposures of Omicron versus Delta reinfection cases—Odds ratios.

Characteristic	OR^1^	95% CI^1^	p-value
Local Health Board			
Aneurin Bevan UHB	—	—	
Betsi Cadwaldr University Health Board	0.77	0.46, 1.28	0.3
Cardiff and Vale University Health Board	0.89	0.51, 1.53	0.7
Cwm Taf Morgannwg University Health Board	0.89	0.50, 1.58	0.7
Hywel Dda University Heath Board	1.00	0.52, 1.99	>0.9
Swansea Bay University Health Board	0.74	0.43, 1.26	0.3
Powys Teaching Health Board	1.36	0.38, 8.67	0.7
Age Group			
0–24	—	—	
25–59	0.54	0.37, 0.78	0.001
60+	0.26	0.14, 0.47	<0.001
Vaccinated			
No	—	—	
1 dose	1.19	0.65, 2.36	0.6
2 doses	1.96	1.33, 2.87	<0.001
3 doses	6.00	1.19, 110	0.09
Unknown	0.58	0.26, 1.50	0.2
Travel			
No	—	—	
Yes	1.23	0.43, 5.22	0.7

^1^OR = Odds Ratio, CI = Confidence Interval

## Discussion

The present study revealed that among those genomically confirmed to be reinfected with Omicron up to February 2022 in Wales, a lower proportion had an initial Wild-type infection, and a higher proportion had an initial Delta infection compared to those reinfected with Delta. Further, the median inter-infection interval was significantly shorter in the Omicron compared to the Delta reinfection group. The median inter-infection intervals reported here are similar to a median reinfection interval of 201 days reported previously in a study covering the period of the emergence and spread of the Alpha variant [15]. The median inter-infection interval we report here is, however, shorter for those reinfected with Omicron compared to Delta (192 versus 226 days, respectively). This is consistent with an increased potential for Omicron to escape immunity specific to older variants [23]. The distribution of the intervals reported here was also more strongly right-skew among the Omicron reinfection group. Presumably, this increased right-skew also reflects the later emergence and dominance of Omicron in Wales, so that the maximum possible inter-infection interval is necessarily longer than that for individuals reinfected with Delta.

Our conditional Poisson model also estimated that the Omicron variant is associated with approximately 2.17 times more reinfections than the Delta variant, when other factors including reinfection by sample date, age and vaccination status are accounted for. This finding corroborates other recent studies reporting higher reinfection rates associated with Omicron relative to the Delta variant [41, 42]. This finding is also consistent with [20] who report that Omicron was associated with a 5.41 fold higher relative risk of reinfection compared to the Delta variant. The current study builds on this work by using cases which have been confirmed through whole genome sequencing as well as genotyping. The findings from the present study also align with analysis of reinfection risk in South Africa by [19] who report an increase in the hazard ratio for reinfection versus primary infection.

This increased reinfection rate is also interesting in the context of a recent sero-surveillance study covering the current study period, in which residual samples from blood donated by Welsh blood donors showed a clear increase in the proportion of samples in which antibodies to the nucleocapsid antigen (anti-N; associated with natural infection) were detected, following the emergence of the SARS-CoV-2 Omicron variant [43]. This is consistent with the increased transmissibility of the Omicron variant and the large wave of cases it caused in the UK [44], but the resulting increase in the cumulative number of first SARS-CoV-2 infections also necessarily increased the size of the pool of individuals who were subsequently ‘eligible’ for reinfection. [43] also showed an earlier and even more rapid increase in the seroprevalence of antibodies to the spike antigen (anti-S), which are associated with vaccination as well as natural infection. Indeed, data as at mid-2022 indicated a high level of COVID-19 vaccination coverage across demographic groups in Wales [45]. Taken together, those findings imply a high level of population immunity gained from prior SARS-CoV-2 exposure by the end of the present study period, particularly for those with hybrid immunity, i.e., immunity gained from a combination of prior natural infection and vaccination. Indeed, country-level studies have shown increased protection from reinfection associated with hybrid relative to natural immunity [46]. We nevertheless found a relatively large number of Omicron reinfections towards the end of the present study period, consistent with the increased capability of Omicron to escape immunity acquired from prior natural infection and vaccination, owing in part to the large number of mutations in the viral spike protein [25, 47–49].

In the present sample, the odds of reinfection with Omicron as opposed to Delta was lower in those aged either 25–59 or 60+ compared to those aged 0–24. This aligns with several prior studies reporting that reinfections are generally more commonly reported in younger individuals [41, 50–52]. propose several potential explanations for this general age-related pattern of reinfection including: 1) younger individuals being potentially less likely to engage in social distancing, and/or more likely to engage in group work and leisure activities, resulting in repeat exposures and reinfections [53], 2) younger individuals are less likely to be vaccinated and therefore more susceptible to reinfection [54], 3) younger individuals are less likely to die of an initial infection and are therefore able to experience reinfection at a higher rate [55], and 4) young individuals have comparatively higher rates of asymptomatic and symptomatic infection which may confer less protection against reinfection [56]. Other studies have, however, found a non-linear U-shaped relationship between age and reinfection rates/risk, with one reporting the reinfection rate was highest in individuals aged 18–29 [57], with this pattern attributed to working-age adults having more frequent social and inter-generational interactions, leading to a higher transmission rate [32, 47]. Extending this previous work, in the present sample, the odds of reinfection with Omicron *relative to* Delta was found to be lower in those aged either 25–59 and 60+ compared to those aged 0–24. This may indicate an interaction between the general age-group differences noted above and the increased transmissibility and reinfection risk posed by the Omicron variant (e.g., the increased risk of reinfection associated with young individuals engaging in more frequent group social interactions, may be exacerbated in the context of a variant with increased immune escape potential). This finding is similar to [20] who reported that older age groups were less likely to be infected with Omicron relative to Delta compared to individuals aged 18–29. As they used narrower age bands than those applied in the present study, however, they also found that individuals aged 0–12 or 13–17 were also less likely to be infected with Omicron relative to Delta than those aged 18–29.

Additionally, the odds of reinfection with Omicron as opposed to Delta, was higher for individuals who had received 2 doses of vaccine by the time of reinfection, compared to those who were not vaccinated. This finding may reflect the later emergence of the Omicron variant. In the context of the staged UK COVID-19 vaccine rollout schedule, more individuals had had the opportunity to receive the second of their two primary vaccine doses by late 2021 when Omicron was causing an increase in the number of initial and reinfections [58].

The high number of infections and reinfections coinciding with the dominance of new variants that is illustrated in Figs 1 and 2 (particularly in the case of Omicron), should not be considered sufficient to suggest an increased risk or incidence of reinfection with emerging variants owing to a number of additional temporally varying variables. Note for instance that in addition to reinfection group differences in characteristics such as age and vaccination status, there was also necessarily a gradually increasing pool of individuals within the population that were ‘eligible’ for reinfection following an initial infection. Testing capacity, and by extension, genome sequencing and genotyping capacity, also increased over the study period, both of which would influence incidence of recorded reinfections, potentially introducing additional biases [59]. We also noted a difference in the proportion of Delta versus Omicron reinfection samples originating from NHS and lighthouse labs; owing to the small number of Delta reinfection cases reported here we did not stratify this finding further by local health board but regional differences in testing policy, reflected in the proportion of samples sequenced across these lab networks, could also potentially introduce biases. Taken together with the development of testing strategies both within and across countries over the course of the pandemic [60–62], this also limits the usefulness of direct comparisons of reinfection rates across countries/studies. The use of the conditional Poisson model to compare Delta and Omicron reinfections in the present study, however, implicitly accounts for some of the additional time-varying variables by comparing reinfections between these groups within model strata that each cover a particular sample collection date. Each strata therefore covers a sufficiently short period of time that for practical purposes, testing policy and capacity etc., can be considered to be matched for Delta and Omicron reinfections occurring in each strata. Work is ongoing to implement this model for real time surveillance of new SARS-CoV-2 variants in Wales. This is only possible due to the high genotyping/sequencing rate in Wales which saw ~29% of all positive Welsh SARS-CoV-2 samples genotyped and/or sequenced over the study period.

### Limitations and future directions

A relatively small number of reinfection cases were available for inclusion in our analyses, particularly as regards Delta reinfections. The number of reinfection cases available for analysis could be substantially increased by pooling genomic data obtained from residents in Wales and other UK nations. Differences in reinfection incidence across the Delta and Omicron groups could then potentially be estimated with greater precision, albeit at the cost of reduced geographical and population specificity. This would also afford inclusion of additional explanatory variables and interactions in the models fitted here. The type of vaccines previously received by reinfection cases could be one such potentially interesting additional factor. However, early evidence supported the efficacy of a ‘mix-and-match’ vaccination schedule, enabling administration of different types of vaccine to individuals across successive doses in the UK [63, 64]. Further studies considering this factor would therefore need to accommodate a potentially large number of vaccine-type combinations.

A related limitation of PCR testing and sequencing data, generally, is that it reflects known and detected infection events, but cannot capture valid infection or reinfection episodes that were not tested and/or reported. There is therefore under-ascertainment of both the original number of cases and the true number of reinfections. There is also no systematic testing for asymptomatic infections, and where asymptomatic testing has occurred it has been in specific cohorts over limited periods of time (e.g., hospitals). Furthermore, different variants may be associated with differential symptomatic infection rates, which could interact with changes in testing policy/practice over time, in turn skewing the representation of particular variants within our sequencing data sample. Taken together, it is possible that the reinfection patterns observed in our sample may not be representative of real-world reinfections. The absence of ground-truth data about the total number real-world infections and the responsible variants, renders this issue challenging to address. Future work could, however, explore the correspondence the number of genomically confirmed reinfections, and antibody/swab positivity results from seroprevalence surveys, as well as the total number of reported cases. This could provide further evidence for the potential for emerging variants to cause further waves of cases and reinfections.

Given the available genomic data, an individual was identified as having had a reinfection if they had a second positive PCR sample recorded 42 days or more after an initial positive sample, both of which were sequenced or genotyped. Due to limited capacity, the number of sequenced and genotyped samples represents a minority of the total number of reported cases (see Fig 1). It is therefore possible that some individuals in the present sample had additional infections prior to or between the two that were here presumed to be their first and second infections. The number of such cases is likely to be small as reinfections were reportedly uncommon during the first two years of the pandemic [18]. Nevertheless, further work could leverage additional testing data to identify and remove such cases.

Finally, the present analyses compared Delta and Omicron reinfections up to February 2022. In principle, a natural extension could be to compare their sublineages. There were, however, relatively few Delta reinfection cases confirmed by either whole genome sequencing or genotyping, rendering further stratification by sublineage unfeasible. Further studies could compare Omicron sublineages though the variant had not diversified substantially by the end of February 2022, so it would be beneficial for such studies to include data from samples received after this date. There were, however, several changes to testing policy in Wales after this date and accounting for these would impact on the number of events in our conditional Poisson model strata.

## Conclusion

This study found that the median interval between first and second infections was shorter for those reinfected with the Omicron rather than the Delta variant of SARS-CoV-2, in the present sample. The Omicron variant was also associated with a higher incidence rate when holding fixed other factors such as age, area of residence and vaccination status, consistent with an increased risk of reinfection. Notwithstanding the challenges of comparing reinfections across emerging variants under conditions of data sparsity and other factors that may reduce generalizability (e.g., variation in number of cases across waves, and uncertainty about the proportion of symptomatic versus asymptomatic cases), the present findings highlight the value of monitoring emerging variants of SARS-CoV-2 that have the potential for causing further waves of cases.
